# Special Significance of Non-*Drosophila* Insects in Aging

**DOI:** 10.3389/fcell.2020.576571

**Published:** 2020-09-22

**Authors:** Siyuan Guo, Xianhui Wang, Le Kang

**Affiliations:** ^1^State Key Laboratory of Integrated Management of Pest Insects and Rodents, Institute of Zoology, Chinese Academy of Sciences, Beijing, China; ^2^CAS Center for Excellence in Biotic Interactions, University of Chinese Academy of Sciences, Beijing, China

**Keywords:** non-*Drosophila* insects, aging, phenotypic plasticity, flight, diapause

## Abstract

Aging is the leading risk factor of human chronic diseases. Understanding of aging process and mechanisms facilitates drug development and the prevention of aging-related diseases. Although many aging studies focus on fruit fly as a canonical insect system, minimal attention is paid to the potentially significant roles of other insects in aging research. As the most diverse group of animals, insects provide many aging types and important complementary systems for aging studies. Insect polyphenism represents a striking example of the natural variation in longevity and aging rate. The extreme intraspecific variations in the lifespan of social insects offer an opportunity to study how aging is differentially regulated by social factors. Insect flight, as an extremely high-intensity physical activity, is suitable for the investigation of the complex relationship between metabolic rate, oxidative stress, and aging. Moreover, as a “non-aging” state, insect diapause not only slows aging process during diapause phase but also affects adult longevity during/after diapause. In the past two decades, considerable progress has been made in understanding the molecular basis of aging regulation in insects. Herein, the recent research progress in non-*Drosophila* insect aging was reviewed, and its potential utilization in aging in the future was discussed.

## Introduction

Aging is regarded as the greatest risk factor of most chronic pathological conditions (Kennedy et al., 2014), and becoming a socioeconomic problem worldwide (He et al., 2016). Between 2000 and 2050, the percentage of population aged above 60 years is projected to increase from approximately 11% to 22% worldwide (United Nations, 2017). As the aging population rapidly grows, aging-related chronic conditions contribute to the biggest proportion of global healthcare burden, and they are estimated to become the next global public health challenge (World Health Organization, 2017). Thus, understanding of aging mechanisms and identifying aging regulators are becoming increasingly important.

Aging is an extraordinary complex process with a time-dependent loss of structure, function, and physiological integrity (Lopez-Otin et al., 2013). Nine molecular aging hallmarks and seven pillars of aging mechanisms have been characterized, including dysfunction or alterations in metabolism, inflammation, stress adaptation, proteostasis, intercellular communication, mitochondrial functions, telomere state, genomic stability, and epigenetics (Lopez-Otin et al., 2013; Kennedy et al., 2014). Most of the current knowledges about aging mechanisms were contributed by canonical model organisms, including yeast (*Saccharomyces cerevisiae*), worm (*Caenorhabditis elegans*), fruit fly (*Drosophila melanogaster*), and house mouse (*Mus musculus*). Fruit fly is a canonical insect model with advantages of rapid life cycle, high fecundity, convenient and precise genetic manipulation, and easy maintenance (Helfand and Rogina, 2003). Studies on fruit fly aging made remarkable contributions to the understanding of conserved aging-regulatory mechanisms, such as endocrine regulation (Toivonen and Partridge, 2009), oxidative stress (Le Bourg, 2001), epigenetic alterations (Solovev et al., 2018), mitochondrial dysfunctions (Guo, 2012), and genomic instability (Li et al., 2013). Moreover, *Drosophila* contains approximately 70% of known disease-related genes in humans (Reiter et al., 2001). Thus, *Drosophila* has been widely used in modeling aging-related diseases of humans and screening for anti-aging drugs (Piper and Partridge, 2018). However, only focusing on a few number of model species ignores the diversity of longevity and aging traits that have evolved in nature, and the diversity provides an opportunity to study various regulators and mechanisms involved in aging plasticity and senescence evolution (Valenzano et al., 2017). Therefore, more non-canonical systems are required for deep understanding of aging biology.

**FIGURE 1:**
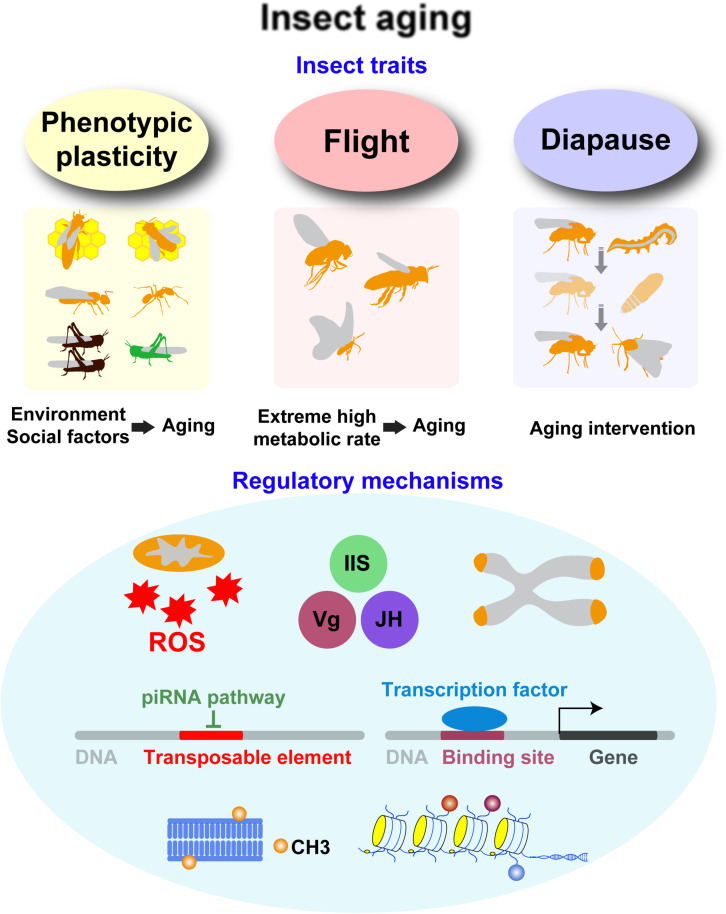
Overview of insect traits with aging-regulatory functions and major molecular mechanisms underlying the effects of these insect traits. Phenotypic plasticity, flight, and diapause have effects on aging. The representative insects and the research implications are shown. Several factors, such as oxidative stress, endocrine factors, telomere, transposable elements, transcription factors, and epigenetics, play important roles in the effects of these insect traits.

As the most diverse group of living animals (Mayhew, 2007), insects have the characteristics of phenotypic plasticity, flight, and diapause (Figure 1), which are considerably essential for aging studies. With single genotype, the lifespan of polyphenic insects, especially social insects, can substantially vary depending on the environment (Keller and Jemielity, 2006), thereby providing an opportunity to study the effects of environmental and social factors on aging. Insect flight achieves the highest metabolic rate known (Kammer and Heinrich, 1978), and excessive oxidative stress associated with hyperactive respiratory metabolism may be the potential aging accelerator (Finkel and Holbrook, 2000). In addition, a special stage of developmental arrest called diapause has evolved in many insect species, enabling them to survive extreme conditions, such as winter (Denlinger, 2002). Diapause results in low metabolic activity and a profound extension of insect lifespan, thereby providing an opportunity to understand the mechanism underlying lifespan extension (Denlinger, 2008; Hahn and Denlinger, 2010).

In the past two decades, advances in genomics, genetic manipulation, and gene editing technology enable the aging studies to approach the multiple phenotypes and molecular levels in the non-*Drosophila* insects. Considerable progress has been achieved in explaining these fantastic aging traits in insects. Here, the recent advances in aging studies of non-*Drosophila* insects were discussed, and the special values of insects as model systems for aging biology were highlighted.

## Phenotypic Plasticity and Aging in Insects

Studies conducted in twins demonstrated that approximately 25% of the variation in human longevity is due to genetic factors, while the rest is due to individual behavior and environmental factors (Herskind et al., 1996; Sebastiani and Perls, 2012). The studies on diet, exercise, chemical exposure, and social connection all demonstrate to affect aging and lifespan in humans through complex and largely unknown mechanisms (Lopez-Otin et al., 2016; Yang et al., 2016; Krutmann et al., 2017; Duggal et al., 2018). Understanding the mechanisms underlying the effects of modifiable environments on aging could help develop treatments to promote human health span.

Many insects have evolved the ability of one genotype to produce more than one alternative phenotype when exposed to different environments (Whitman and Ananthakrishnan, 2009; Simpson et al., 2011). Polyphenic insects offer striking examples of natural variation in longevity, such as reproductives and workers in social insects, gregarious and solitary locusts, spring and summer butterflies, and winged and wingless aphids (Keller and Jemielity, 2006; Pener and Simpson, 2009; Ogawa and Miura, 2014; Freitak et al., 2019). Unlike canonical model species, polyphenic insects exhibit up to 100-fold changes in longevity in response to environmental changes (Remolina and Hughes, 2008), revealing their specific value in studying the effects of environmental factors on aging. Moreover, a number of environmental factors are involved in regulating the phenotypic plasticity of insects (Whitman and Ananthakrishnan, 2009; Simpson et al., 2011). Diet, reproduction, behavior, social interaction, population density, photoperiodic cues, and temperature contribute to lifespan variations in polyphenic insects (Keller and Jemielity, 2006; Pener and Simpson, 2009; Ogawa and Miura, 2014; Freitak et al., 2019). Therefore, the extreme lifespan differences and enormous influencing factors provide an opportunity to deeply understand the mechanisms behind aging plasticity induced by complex environmental changes.

The differences in the lifespan of divergent morphs of butterflies, aphids, and locusts are suitable samples for studying the effects of photoperiodic cues and temperature, environmental stress, and population density on aging, respectively (Pener and Simpson, 2009; Ogawa and Miura, 2014; Freitak et al., 2019). Although previous studies have discovered some interesting aging characteristics, such as key roles of population density at the early life on locust aging (Boerjan et al., 2011) and the positive relationship between immune activity and longevity in seasonally polyphenic butterfly (Freitak et al., 2019), the mechanisms remain unknown.

## Social Insects in Aging

Social insects such as honey bees, bumble bees, ants, and termites, represent the ideal model systems to investigate the mechanisms behind the effects of social factors on aging because of the enormous intraspecific variation in their lifespan and aging rate (Keller and Jemielity, 2006; Kramer et al., 2016). Highly reproductive honeybee queens can survive for two years, whereas sterile workers can only survive between few weeks and one year (Seeley, 1978; Winston, 1987). The reproductives of social ants and termites can live up to 30 years (Hölldobler and Wilson, 1990), while workers frequently have 10-fold shorter lifespans (Kramer and Schaible, 2013). Moreover, the aging rate and lifespan of workers considerably vary depending on environmental and task changes. For example, the tasks of honeybee workers change in an orderly and usually age-dependent manner, with young workers performing nursing duties and old ones foraging (Winston, 1987). The timing of transition from in-hive tasks to foraging is the most significant predictor of worker lifespan, because foragers senesce faster than their same-aged nurse counterparts (Seehuus et al., 2006a; Behrends et al., 2007; Münch et al., 2008; Quigley et al., 2018). Honeybee workers can revert from foraging duties to hive activities, and this reversion is associated with the reversal of aging biomarkers (Amdam et al., 2005; Baker et al., 2012; Herb et al., 2012). Some eusocial ants possess a type of social organization that enables adult workers to become reproductive individuals or gamergates following removal of queen (Brunner et al., 2011). With the reallocation of tasks, the fertile workers could achieve an extended lifespan (Hartmann and Heinze, 2003; Schneider et al., 2011; Kohlmeier et al., 2017).

Several mechanisms are involved in regulating caste-specific aging rate in social insects. First, the difference in antioxidant capacity is one of the putative reasons. The level of vitellogenin (Vg), which protects organisms from oxidative stress (Seehuus et al., 2006b), is higher in honeybee individuals with longer lifespan; for instance, it is higher in queens than in workers and higher in nurses than in foragers (Amdam et al., 2005; Corona et al., 2007; Münch and Amdam, 2010). Polyunsaturated fatty acids that are high in pollen but negligible in royal jelly may result in the cellular membrane of honeybee workers becoming more susceptible to lipid peroxidation than that of queens (Haddad et al., 2007; Martin et al., 2019). However, the role of antioxidant genes in queen-biased longevity is controversial. Some studies on honeybees and ants revealed a lower level of antioxidant genes in reproductives than in workers (Parker et al., 2004; Corona et al., 2005; Schneider et al., 2011). Second, endocrine factors also play key roles in caste-specific aging phenotypes. The interaction between insulin/IGF (insulin-like growth factor)-like signaling (IIS), juvenile hormone (JH), and Vg jointly regulates longevity and reproduction (Rodrigues and Flatt, 2016). However, the aging-regulatory functions of endocrine network are not conserved across social insects. For example, unlike the fire ant and several primitively eusocial insects (Robinson and Vargo, 1997; Bloch et al., 2002), in honeybee workers, JH appears to be life-shortening hormone because JH and Vg are configured in a mutually repressive regulatory circuitry during adult stage (Guidugli et al., 2005; Page and Amdam, 2007). Less conserved interaction pathways between endocrine factors (e.g., microRNAs) may contribute to species variation in the longevity regulation of endocrine network (Marco et al., 2010; Nunes et al., 2013). Third, the difference in the maintenance of genomic stability and telomere may be one of potential mechanisms. The heads of termite reproductives, not the major workers, prevent aging-related genomic damages caused by transposable element activity through continued upregulation of the piRNA pathway (Elsner et al., 2018). Telomerase activity displays a 70-fold increase in brains of adult honeybee queens compared to those of adult workers (Korandová and Frydrychová, 2016). The piRNA pathway and telomerase being primarily in germline suggests that the reproductives of highly social insects could be regarded as equivalent to germline of a colony, whereas the workers are equivalent to disposable soma (Elsner et al., 2018). The reproductives have evolved germline-corresponding anti-aging mechanisms to sustain themselves through generations. The fourth potential mechanism is epigenetic regulation. Caste-specific methylation profiles are associated with conserved aging-regulatory pathways, including IIS components, IIS-related metabolic systems, JH-responsive genes, and telomere maintenance (Bonasio et al., 2012; Foret et al., 2012), suggesting that DNA methylation may contribute to the aging differences between queens and workers. Genomic demethylation by pharmacological inhibition increases Vg expression and extends the lifespan of worker bees (Cardoso-Júnior et al., 2018), indicating the potential roles of DNA methylation in regulating aging in workers.

Although considerable progress has been achieved in understanding the mechanism behind aging divergences in social insects, research evidence remains lacking in some important related issues. Many aging-regulatory genes display differences at the transcriptional level between castes in social insects, suggesting that transcriptional regulation plays crucial roles in caste-specific aging trajectories. Epigenetics connects environmental inputs with transcription and thus may be the key to the aging differences between castes (Benayoun et al., 2015). However, the interplay between many types of epigenetic mechanisms and caste-specific aging is rarely studied, although the crucial roles of histone modifications and microRNAs in establishing caste-specific transcriptional programs and caste differentiation have been proposed (Weaver et al., 2007; Bonasio et al., 2010; Spannhoff et al., 2011; Guo et al., 2013; Simola et al., 2013, 2016; Shi et al., 2015; Ashby et al., 2016; Wojciechowski et al., 2018).

## Flight and Aging in Insects

Metabolic rates may be related to aging and longevity. The rate of living theory proposed at the beginning of the 20th century suggests that a slowed rate of metabolism is associated with lengthened longevity (Rubner, 1916). In line with this view, recent studies revealed that increased resting metabolic rate is a risk factor for mortality in humans (Ruggiero et al., 2008; Jumpertz et al., 2011). High metabolic rates have been hypothesized to come with a cost in terms of increased level of reactive oxygen species (ROS), the byproducts of mitochondrial metabolism, which leads to accelerated aging through damaging macromolecules, including DNA, lipid, and proteins (Harman, 1956; Finkel and Holbrook, 2000). Data from humans revealed that changes in metabolic rates are accompanied by changes in oxidative stress and may underlie variation in aging rate (Redman et al., 2018). However, the relationship between metabolic rate, ROS, and lifespan is highly complex. The positive relationship between metabolic rate and ROS production provokes great debate (Speakman, 2005), and the beneficial roles of ROS in regulating lifespan, metabolism, and development have been demonstrated (Santos et al., 2018). The true mechanisms of the association between metabolic rates and aging are not well understood.

Insect flight has the highest metabolic rate (Kammer and Heinrich, 1978) and profound effects on aging process. In line with the early metabolic and locomotor senescence in *Drosophila* after forcing flight (Lane et al., 2014), aging is accelerated in honeybee workers after transitioning from infrequently flying nurses to frequently flying foragers (Seehuus et al., 2006a; Behrends et al., 2007). Moreover, foraging bees with flight restriction do not display aging-related learning deficits as the free-flying ones (Tolfsen et al., 2011). Flight restriction similarly decreases the mitochondrial damage and extends lifespan to approximately threefold of the normal in houseflies (Agarwal and Sohal, 1994; Yan and Sohal, 2000). These results collectively implicated the negative effects of flight on insect aging. However, flight experience is not always detrimental. For instance, flight restriction leads to increased oxidative damage in brains of honey bees and early senescence of flight performance in fruit flies (Tolfsen et al., 2011; Lane et al., 2014). A high flight activity rate within the activity days has no negative effects on longevity in two bee species in the fields (Straka et al., 2014). In Glanville fritillary butterfly (*Melitaea cinxia*), peak flight metabolic rates are positively associated with lifespan (Niitepõld and Hanski, 2013). Flight treatment alone has no effect on the longevity of some butterflies, including Glanville fritillary butterfly (Woestmann et al., 2017), Mormon fritillary (*Speyeria mormonia*) (Niitepõld and Boggs, 2015), speckled wood butterfly (*Pararge aegeria*) (Gibbs and Van Dyck, 2010), and squinting bush brown butterfly (*Bicyclus anynana*) (Saastamoinen et al., 2010). Therefore, the effects of flight behavior on insect longevity and aging seem to vary depending on species, flight traits, physiological states, and some other factors.

Elevated oxidative stress is considered as the primarily mechanism underlying the negative effects of flight on lifespan. In insects, flight could induce oxidative stress by increasing ROS generation from respiratory metabolism and altering membrane lipid composition that is more susceptible to ROS (Sohal et al., 1984; Yan and Sohal, 2000; Magwere et al., 2006; Williams et al., 2008; Margotta et al., 2018). Elevated oxidative stress in insects is deleterious in most cases. An increase in oxidative stress by pharmacological and genetic manipulations shortens lifespan in some insects (Phillips et al., 1989; Parkes et al., 1998; Kirby et al., 2002; Duttaroy et al., 2003; Cui et al., 2004; Margotta et al., 2018). However, the mechanism underlying the non-negative effects of insect flight on lifespan has not yet been studied. One potential explanation for these effects is that the oxidative stress generated by moderate flight may induce long-term stress response, thus protecting organisms from damage accumulation (Gems and Partridge, 2008). Another possible explanation is that some insects may evolve specific antioxidant mechanisms. For example, to resist oxidative stress during hovering flight, the tobacco hornworm (*Manduca sexta*) fed with nectar sugar generated antioxidant compounds by shunting glucose via low-energy pentose phosphate pathways (Levin et al., 2017). In addition, ROS does not always play negative roles in insect longevity; it could extend longevity by inducing diapause in cotton bollworm (*Helicoverpa armigera*) (Zhang et al., 2017).

Studies on the mechanisms behind the effects of insect flight on aging mainly focused on oxidative stress, and few studies on other aging-regulatory mechanisms are available. Insect flight induces substantial changes in endocrine status and gene expression (Rademakers and Beenakkers, 1977; Goldsworthy, 1983; Margotta et al., 2013; Kvist et al., 2015; Woestmann et al., 2017). Whether and how these endocrine and transcriptional changes influence aging process remain elusive. Moreover, insect flight experience influences oxidative damage in a tissue-dependent manner (Williams et al., 2008; Margotta et al., 2013, 2018). Flight-susceptible tissues may further affect systemic aging through inter-tissue crosstalk (Demontis et al., 2013). However, the key tissues and signals involved are still unknown. Lastly, insect species vary widely in flight traits, such as wingbeat frequency, flight duration, and wing morphology (Molloy et al., 1987; Dudley, 2002). Such variations in flight traits have effects on the differences in flight metabolic properties (Casey et al., 1985; Feller and Nachtigall, 1989; Darveau et al., 2014), and they may be involved in species variation in the effects of flight on aging through unknown mechanisms.

## Diapause and Aging in Insects

How to extend lifespan has always fascinated people throughout human history. Science fictions depict that humans extend lifespan and reach the future through achieving hypometabolic states and cryonics. Interestingly, this specific ability is common in insects. Diapause, a state of programmed arrest of development coupled with suppressed metabolic activity, helps insects to survive unfavorable environmental conditions (Denlinger, 2002). During diapause, insects do not experience the same fast “aging clock” as in direct development, resulting in drastically extended lifespan (Tatar and Yin, 2001). Moreover, insects have evolved diapause at different life cycle stages, including eggs, larvae, pupae, and adults, thereby providing opportunities to study the effects of various diapause types on aging (Denlinger, 2002). Studying insect diapause could provide new insights into aging interventions and lifespan extension (Denlinger, 2008).

Insect systems demonstrate organismal and genetic links between diapause and aging. Similar with *Drosophila*, which undergoes a negligible senescence during reproductive diapause (Tatar et al., 2001a,b), adult monarch butterflies and grasshoppers with reproductive diapause induced by surgical removal of the *corpora allata* have doubled lifespan (Herman and Tatar, 2001; Tatar and Yin, 2001). In addition, a handful of evidence revealed the roles of pupal and larval diapauses on the extension of pre-adult longevity (Lu et al., 2013; Lin and Xu, 2016; Lin et al., 2016; Zhang et al., 2017; Wang et al., 2018). Noteworthy, diapause not only slows aging during diapause phase, but also has species-dependent effects on adult longevity after diapause. For example, maize stalk borer (*Busseola fusca*) and spotted stem borer (*Chilo partellus*) have shortened adult lifespans after diapause (Gebre-Amlak, 1989; Dhillon and Hasan, 2018), but cotton bollworm (*H. armigera*) and multivoltine bruchid (*Kytorhinus sharpianus*) display extended lifespans after diapause (Ishihara and Shimada, 1995; Chen et al., 2014).

Transcriptional regulation may play a crucial role in diapause-related aging regulation (Denlinger, 2002). Several key transcription factors involved have been characterized. In the mosquito *Culex pipiens*, transcription factor FoxO, which is regulated by insulin and JH signaling, alters the expression of aging-regulatory genes during diapause (Sim and Denlinger, 2008, 2013a,b; Sim et al., 2015). In the moth *H. armigera*, accumulation of FoxO induced by high ROS activity during diapause also promote lifespan extension (Zhang et al., 2017). The diapause-related ROS increase is attributed to the downregulation of hexokinase expression, which is regulated by transcription factors CREB, c-Myc, and POU (Lin and Xu, 2016). In addition, repression of mitochondrial activity, which may be related to lifespan extension of diapause, is regulated by a network of transcription factors HIF-1α, CREB, Smad1, POU, and TFAM (Lin et al., 2016; Li et al., 2018; Wang et al., 2020). Except for transcription factors, epigenetic mechanism may also influence the transcriptional alterations of aging-regulatory genes during diapause (Reynolds, 2017). Studies have proposed that DNA methylation (Pegoraro et al., 2016), histone modifications (Lu et al., 2013; Hickner et al., 2015; Sim et al., 2015; Reynolds et al., 2016), non-coding RNAs (Reynolds et al., 2013, 2017; Poupardin et al., 2015; Yocum et al., 2015; Reynolds, 2019), and RNA methylation (Jiang et al., 2019) may all contribute to diapause-related transcriptional changes and phenotypes. The phenomenon of extended adult lifespan after diapause in some insects suggests that the expression levels of aging-regulatory genes persist after diapause termination. The possible cause of species variation in this phenomenon is species-specific transcriptional maintenance. The transcriptional regulatory mechanisms underlying diapause seem to vary across insects. Interspecific comparisons revealed little transcriptional similarity among diapauses across invertebrates (Ragland et al., 2010). DNA methylation play roles in diapause regulation in the wasp *Nasonia vitripennis* but not in the silkmoth *Bombyx mori* (Pegoraro et al., 2016; Yuichi et al., 2016).

Some gaps exist in understanding the effects of diapause on aging. Although a great variation in the expression of aging-related genes during diapause has been documented, experimental evidence of cause-and-effect relationships between gene expression and aging is still lacking. Moreover, whether the expression levels of these diapause-induced aging-regulatory genes persist after diapause termination and the underlying mechanisms involved are unclear.

## Conclusion and Perspective

Substantial progress has been achieved in enhancing the understanding of the molecular basis of aging regulation in insect aging. Obviously, these underlying molecular mechanisms were highly intertwined processes. Transcriptional differences are the most observed differences in aging-regulatory genes involved in endocrine regulation, oxidative stress responses, maintenance of telomere, and genomic stability (Amdam et al., 2005; Corona et al., 2007; Bonasio et al., 2010; Elsner et al., 2018). These transcriptional differences may be attributed to variations in transcription factors and epigenetic states, which in turn are influenced by endocrine factors (Sim and Denlinger, 2008, 2013a; Vaiserman et al., 2018). The mechanisms mentioned above also play critical roles in mammalian and human aging (Lopez-Otin et al., 2013). For instance, reduced insulin signaling is related to extended longevity in social insects and mammals (Tatar et al., 2003; Corona et al., 2007; Ament et al., 2008), although the insulin pathways considerably vary across species (Corona et al., 2007; Smýkal et al., 2020). DNA methylation is closely related to aging from insects to mammals (Herb et al., 2012; Yan et al., 2015; Horvath and Raj, 2018), although the differences in genomic DNA methylation between insects and vertebrates are highly significant (de Mendoza et al., 2020). This finding suggests that aging-regulatory pathways are evolutionarily conserved, although the detailed mechanisms may vary across species. Thus, aging studies on non-*Drosophila* insects could expand the understanding of aging regulators and help develop anti-aging interventions. Here, several perspectives for further studies on insect aging are provided as follows.

First, studying the aging mechanisms underlying aging plasticity in non-social insects is highly valuable. Transcription regulation represents one of the key mechanisms underlying aging regulation, and it is the downstream of environment-induced epigenetic changes. Thus, transcriptome analysis could be used to screen key aging genes and pathways underlying aging plasticity in these species.

Second, determining epigenetic mechanisms underlying aging plasticity is essential. A large number of studies suggest that epigenetic factors have potential roles in aging regulation in polyphenic insects. Epigenetic marks are plastic, and many drugs targeting epigenetic enzymes are available (Heerboth et al., 2014). Investigating the link between epigenetic information and environment cues and the epigenetic mechanisms behind insect aging could provide new insights into treatments for aging retardation and reversal.

Third, the effects of insect flight and diapause on aging vary largely depending on insect species. The diversity of insects offers rich resources for cross-species comparisons. Thus, interspecific analysis could help elucidate the mechanisms underlying the beneficial effects of insect flight and diapause on adult longevity, which may reveal new strategies to prevent collapse during aging.

Thousands of insect genomes have been sequenced (Li et al., 2019), and gene editing tools have been developed in various insects (Gantz and Akbari, 2018; Hillary et al., 2020). A strong and growing arsenal of powerful technologies provides a huge support for elucidating the molecular mechanisms underlying insect aging. These novel insect models are expected to result in groundbreaking discoveries and ultimately promote human healthy aging in the future.

## Author Contributions

LK and XW designed the research. SG collected the references. SG, LK, and XW wrote the manuscript. All authors contributed to the article and approved the submitted version.

## Conflict of Interest

The authors declare that the research was conducted in the absence of any commercial or financial relationships that could be construed as a potential conflict of interest.
